# Icariin promotes the proliferation and osteogenic differentiation of bone-derived mesenchymal stem cells in patients with osteoporosis and T2DM by upregulating GLI-1

**DOI:** 10.1186/s13018-023-03998-w

**Published:** 2023-07-15

**Authors:** Sheng-li Xia, Zi-yuan Ma, Bin Wang, Feng Gao, Sheng-yang Guo, Xu-han Chen

**Affiliations:** 1 Department of Orthopedics, Shanghai University of Medicine and Health Sciences Affiliated Zhoupu Hospital, Shanghai, 201318 China; 2 Zhoupu Community Health Service Center, 163 Shenmei East Road, Pudong New Area, Shanghai, 201318 China

**Keywords:** Icariin, T2DM, GLI-1, Osteogenic differentiation, Osteoporosis

## Abstract

**Background:**

The function of mesenchymal stem cells (MSCs) from patients with osteoporosis (OP) is impaired and worsens in patients with type 2 diabetes mellitus (T2DM). Icariin (ICA) is the major active flavonoid glucoside isolated from traditional Chinese herbal *Epimedium pubescens*, and confirmed able to improve bone mass of OP patients.

**Objective:**

To investigate the effect of ICA on the proliferation and osteogenic differentiation of bone-derived MSCs (BMSCs) from patients with OP and T2DM and uncover the potential mechanism.

**Methods:**

BMSCs were treated with ICA, and proliferation and osteogenic potency were evaluated using the 2,5-diphenyl-2H-tetrazolium bromide (MTT) assay and detection of osteogenic markers (ALP, RUNX2, SPP1, COL1A1, and mineralized nodules) was performed. RNA sequencing and bioinformatic analysis were performed to identify differentially expressed genes (DEGs) after ICA treatment and screen proliferation- and osteogenic differentiation-related processes. Gene gain and loss were performed to confirm the role of the key candidate gene.

**Results:**

ICA significantly promoted the proliferation and osteogenic differentiation of BMSCs. A total of 173 DEGs were identified after ICA treatment. Six DEGs (GLI-1, IGF2, BMP6, WNT5A, PTHLH, and MAPK14) enriched in both proliferation- and osteogenic differentiation-related processes were screened; GLI-1 had the highest validated |log2FC| value. Overexpression of GLI-1 enhanced the proliferation and osteogenic differentiation of BMSCs, and knockdown of GLI-1 weakened the positive effect of ICA on BMSCs.

**Conclusion:**

ICA promoted the proliferation and osteogenic differentiation of impaired BMSCs by upregulating GLI-1.

**Supplementary Information:**

The online version contains supplementary material available at 10.1186/s13018-023-03998-w.

## Introduction

Osteoporosis (OP) is a commonly occurring public disease, and the prevalence of OP among individuals over 50 years of age is > 15% in Asia, two-fold higher than that in Europe [1, 2]. OP is characterized by quantitative and qualitative degeneration of bone tissues, causing an increase in fracture risk. However, the fracture risk is further enhanced when patients are diagnosed with type 2 diabetes mellitus (T2DM), even if they have a similar area bone mineral density as non-diabetic controls [3]. T2DM affects > 400 million individuals worldwide, and this figure is increasing with the aging of society [4]. Studies indicate that the cumulative 10-year incidence of fractures in newly diagnosed T2DM patients is > 30% [5], which is closely associated with an increase in skeletal fragility [6]. Skeletal homeostasis is an intricate process predominantly regulated by bone-resorbing osteoclasts and bone-forming osteoblasts, which work together to maintain the balance of bone metabolism. When bone formation is slower than resorption, skeletal fragility increases. Therefore, enhancement of bone formation has been an important research hotspot in the past decades.

Mesenchymal stem cells (MSCs) are a type of undifferentiated cell with self-proliferation and multi-lineage differentiation capabilities that play important roles in bone formation [7]. Generally, MSCs can migrate to the bone marrow and differentiate into pre-osteoblasts in the early stages of bone repair [8]. However, MSCs from OP models show reduced migration ability [9], resulting in a decrease in the number of MSCs, as well as impaired osteogenic differentiation capability [10]. Currently, supplementary MSCs have been developed as a potential treatment for OP and their efficacy has been shown in several animal models [11]. The latest clinical trial result shows that transplantation of MSCs is beneficial to fracture healing by promoting bone architecture, and its safety has also been confirmed in this trial [12]. However, transplantation of MSC for diabetic OP has been less reported. The primary reason for this is that hyperglycemic conditions inhibit proliferative capability, increase apoptosis, and weaken multi-potency, colony-forming efficiency, and osteogenic differentiation capability [13, 14], resulting in further impairment of MSC function. Therefore, amelioration of the proliferation and osteogenic differentiation capabilities of impaired MSC is the foundation for further in vivo transplantation.

Icariin (ICA) is the major active flavonoid glucoside isolated from the herb *Epimedium pubescens*, which has been used for the treatment of OP for decades in Chinese medicine. Studies indicate that ICA can prevent ovariectomy-induced bone loss and reduce femoral and tibial strength [15, 16]. It also promotes the proliferation and osteogenic differentiation capabilities of various normal MSCs [17, 18]. Our previous study obtained several bone-derived MSCs (BMSCs) from patients with OP diagnosed with T2DM, and these cells showed a lower osteogenic differentiation potential than those isolated from non-T2DM patients with OP [19]. We inferred that ICA may also have a positive effect on impaired BMSCs.

**Fig. 1:**
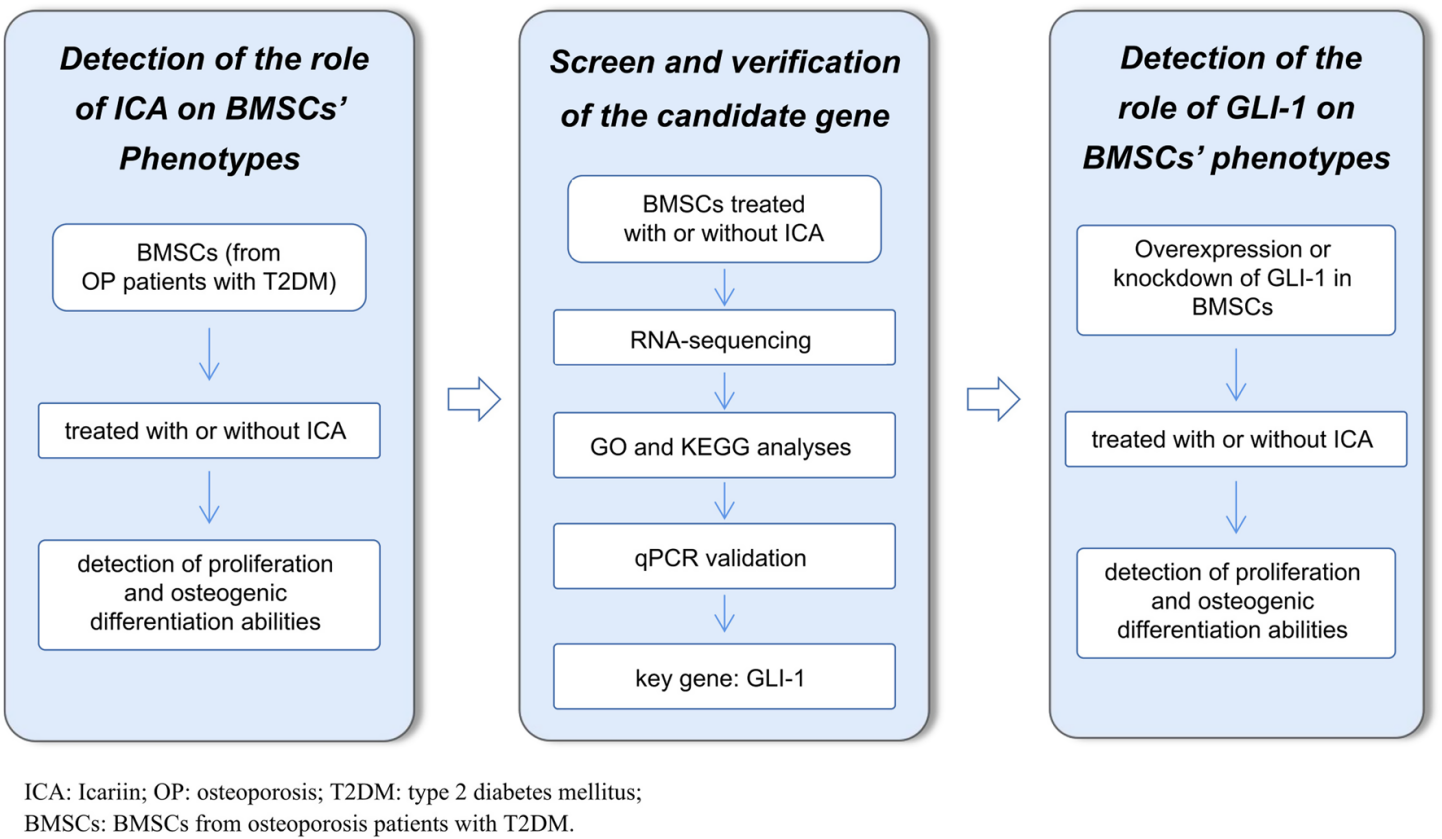
Flow diagram of the study

In the present study, the effect of ICA in proliferation and osteogenic differentiation was investigated, and RNA sequencing and bioinformatic analysis were performed to screen the key effector genes of ICA. Next, GLI family zinc finger 1 (GLI-1), which plays important roles in embryonic development and tumorigenesis [20], was screened. Finally, we showed that ICA ameliorated the function of impaired BMSCs by increasing their expression. The overall design of the study is presented in Fig. 1.

## Materials and methods

### Cells and cell culture

BMSCs were isolated from three patients with OP diagnosed with T2DM (71.33 ± 3.05 years old; F, F, M) in our previous study [19], and cultured in Minimum Essential Medium supplemented with 10% fetal bovine serum and 1% penicillin–streptomycin. Cells were maintained at 37 °C in a 5% humidified CO_2_ atmosphere. The above reagents were all purchased from Gibco (Thermo Fisher Scientific, Waltham, MA, USA).

### Cytotoxicity

BMSCs were seeded in a 96-well plate at a density of 7 × 10^3^ cells/well and incubated overnight. Then, cells were incubated with different concentrations (0, 1, 5, 10, 50, and 100 μM) of ICA (Selleck, Shanghai, China) for 48 h. Three replicates were used for each concentration. Next, the medium was removed and 2,5-diphenyl-2H-tetrazolium bromide (MTT) was added and incubated for an additional 3 h, followed by the detection of the optical density (OD) value at 492 nm. Cell viability (%) was used to reflect cytotoxicity.

### Proliferation assay

Cells were re-suspended at a density of 4 × 10^4^ cells/mL, and 100 μL/well of the cells was seeded into a 96-well plate. ICA (0, 1, 5, and 10 μM) was cultured with cells for 1, 3, and 5 d, and the MTT assay was used to detect cell viability following the above steps. Three replicates were performed for each concentration. Proliferation rate was represented using the ratio of OD value (on day 3,5) to the control OD value (on day 1).

### Detection of alkaline phosphatase (ALP) activity

Cells were seeded in a 12-well plate and cultured to a confluence of approximately 90%. Next, 0.1 mg/mL dexamethasone, 50 μg/mL ascorbic acid, and 10 mM glycerophosphate (all from Sigma-Aldrich) were added and used to induce osteogenic differentiation. ICA (0, 1, 5, and 10 μM) was also added during the induction process. Following induction for 7 d, the activity of ALP was tested using a commercial ALP assay kit (Beyotime, Shanghai, China), and the total protein was detected using a bicinchoninic acid (BCA) kit (Beyotime). ALP activity was presented as the ratio of the OD value at 405 nm per milligram of total protein. All samples were analyzed in triplicate.

### Quantitative polymerase chain reaction (qPCR)

Total RNA was extracted from cells using a universal RNA extraction kit (TAKARA, Dalian, China), following the manufacturer’s instructions. The concentration of RNA was measured on a micro-spectrophotometer, and 1 µg of RNA was then transcribed into complementary DNA (cDNA) using ReverTra ACE (Toyobo Life Science, Osaka, Japan), according to the manufacturer’s protocols. Next, the LightCycler 480 SYBR Green I Master system (Roche Diagnostics, GmbH) was used for quantification. One microliter of cDNA was mixed with 1 µL of primers (10 µM; synthesized by Sangon Biotech Co., Ltd., [Shanghai, China]) and 10 µL of SYBR Green, then the resulting mixture was reacted following the thermal cycling: 95 °C for 10 s, 60 °C for 20 s, and 72 °C for 20 s for 42 cycles. The relative expression of mRNA was calculated using the 2^−ΔΔCt^ method, and the data were normalized to GAPDH. The primer sequences are listed in Table 1. All samples were analyzed in triplicate.

**Table 1:** Primer sequences used for qPCR

Genes	Forward (5'-3')	Reverse (5'-3')
RUNX2	TGGTTACTGTCATGGCGGGTA	TCTCAGATCGTTGAACCTTGCTA
SPP1	CTCCATTGACTCGAACGACTC	CAGGTCTGCGAAACTTCTTAGAT
COL1A1	GAGGGCCAAGACGAAGACATC	CAGATCACGTCATCGCACAAC
GLI1	AGCGTGAGCCTGAATCTGTG	CAGCATGTACTGGGCTTTGAA
IGF2	GTGGCATCGTTGAGGAGTG	CACGTCCCTCTCGGACTTG
BMP6	AGCGACACCACAAAGAGTTCA	GCTGATGCTCCTGTAAGACTTGA
WNT5A	ATTCTTGGTGGTCGCTAGGTA	CGCCTTCTCCGATGTACTGC
PTHLH	ATTTACGGCGACGATTCTTCC	GCTTGGAGTTAGGGGACACC
MAPK14	CCCGAGCGTTACCAGAACC	TCGCATGAATGATGGACTGAAAT
GAPDH	GGAGCGAGATCCCTCCAAAAT	GGCTGTTGTCATACTTCTCATGG

### Alizarin Red S (ARS) staining

After osteogenic induction for 21 d, cells were fixed using 4% paraformaldehyde (Servicebio, Wuhan, China) and then incubated with 1% ARS staining solution (Solarbio, Beijing, China) for 20 min at room temperature. The results of staining were captured using a conventional camera, and the calcified matrices were resolved using 10% cetylpyridinium chloride, followed by the detection of the OD value at 562 nm. All samples were analyzed in triplicate.

### RNA sequencing and pathway analysis

BMSCs were treated with or without 5 µM ICA for 3 d in normal medium and then lysed using Trizol reagent (Invitrogen; Thermo Fisher Scientific). Three replicates were used for sequencing. Total RNA was extracted using an RNeasy mini kit (Qiagen, Germany), following the manufacturer’s instructions, and mRNA molecules were purified using poly T oligo-attached magnetic beads. Next, mRNA was fragmented, followed by the synthesis of the first- and second-strand cDNA. Subsequently, the cDNA fragments underwent an end repair process, and the resulting products were enriched through PCR to create the cDNA library, which was then quantified using a Qubit®2.0 Fluorometer (Life Technologies, USA) and validated using an Agilent 2100 bioanalyzer (Agilent Technologies, USA). Clusters were generated using cBot with a 10 pM library and sequenced using an Illumina NovaSeq 6000 platform (Illumina, USA). Library construction and sequencing were performed by Sinotech Genomics Co., Ltd. (Shanghai, China).

Gene abundance was expressed as fragments per kilobase of exon per million mapped reads (FPKM). Stringtie software was used to quantify the fragments within each gene, and the TMM algorithm was used for normalization. The R package edgeR was used to analyze the differential expression of mRNA. |fold change (FC)| value > 2 and *p* value < 0.05 were used as filters to screen differentially expressed genes (DEGs). Raw data of six samples were deposited in SRA database, and accessible through the SRA Run Accession IDs (SRR24689237, SRR24689238, SRR24689239, SRR24689240, SRR24689241, SRR24689242). Relevant bioproject was accessible in https://www.ncbi.nlm.nih.gov/bioproject/PRJNA975270. Venn diagram analysis was performed to analyze overlapping DEGs among the groups.

Gene Ontology (GO) and Kyoto Encyclopedia of Genes and Genomes (KEGG; http://www.genome.ad.jp/kegg) pathway analyses were performed using the enriching R package. The terms or pathways were ranked in descending order according to the enrichment factor, and the top 30 terms or pathways were selected for visualization.

### Cell transfection

The cells were seeded in 6-well plates and cultured to a confluence of approximately 80%. The overexpression plasmid (pcDNA3.1-GLI-1) and the small interfering RNA (siRNA) targeting GLI-1 were transfected into cells using Lipofectamine® 3000 (Invitrogen), following the manufacturer’s instructions. After transfection for 48 h, qPCR was performed to verify efficacy. The empty plasmid and the negative control (NC) siRNA were also transfected into cells and referred to as the NC and si-NC groups, respectively. The overexpression plasmid and si-GLI-1 sequences were synthesized by RiboBio Co., Ltd. (Guangzhou, China). Three replicates were used for cell transfection.

### Western blotting

Briefly, the cells were lysed using radioimmunoprecipitation assay (RIPA) lysis buffer (Beyotime), and the supernatant was collected after centrifugation. Protein concentration was detected using a BCA kit (Beyotime), following the manufacturer’s instructions. Next, 15 µg of protein was separated using 12% sodium dodecyl-sulfate polyacrylamide gel electrophoresis (SDS-PAGE gels) and transferred to polyvinylidene difluoride (PVDF) membranes, followed by blocking with 5% non-fat milk. Then the membranes were incubated with the primary antibodies (GAPDH and GLI-1) for 12 h at 4 ℃, as well as the secondary antibody for 2 h at room temperature. Finally, the band signals were enhanced using a universal ECL kit (Pierce; Thermo Fisher Scientific), and GAPDH was used as the internal control. All antibodies were purchased from ABclonal Co., Ltd. (Wuhan, China). All samples were analyzed in triplicate.

### Statistical analysis

SPSS 17.0 software (SPSS Inc, Chicago, IL) was used for statistical analysis. Data are shown as the mean ± standard deviation (SD). Unpaired Student's t-test was used to analyze the differences between two groups. One-way analysis of variance (ANOVA) was used for comparisons among multiple groups. The statistical analysis was repeated once. Statistical significance was set at *p* < 0.05.

## Results

### ICA promotes the proliferation and osteogenic differentiation of BMSCs

**Fig. 2:**
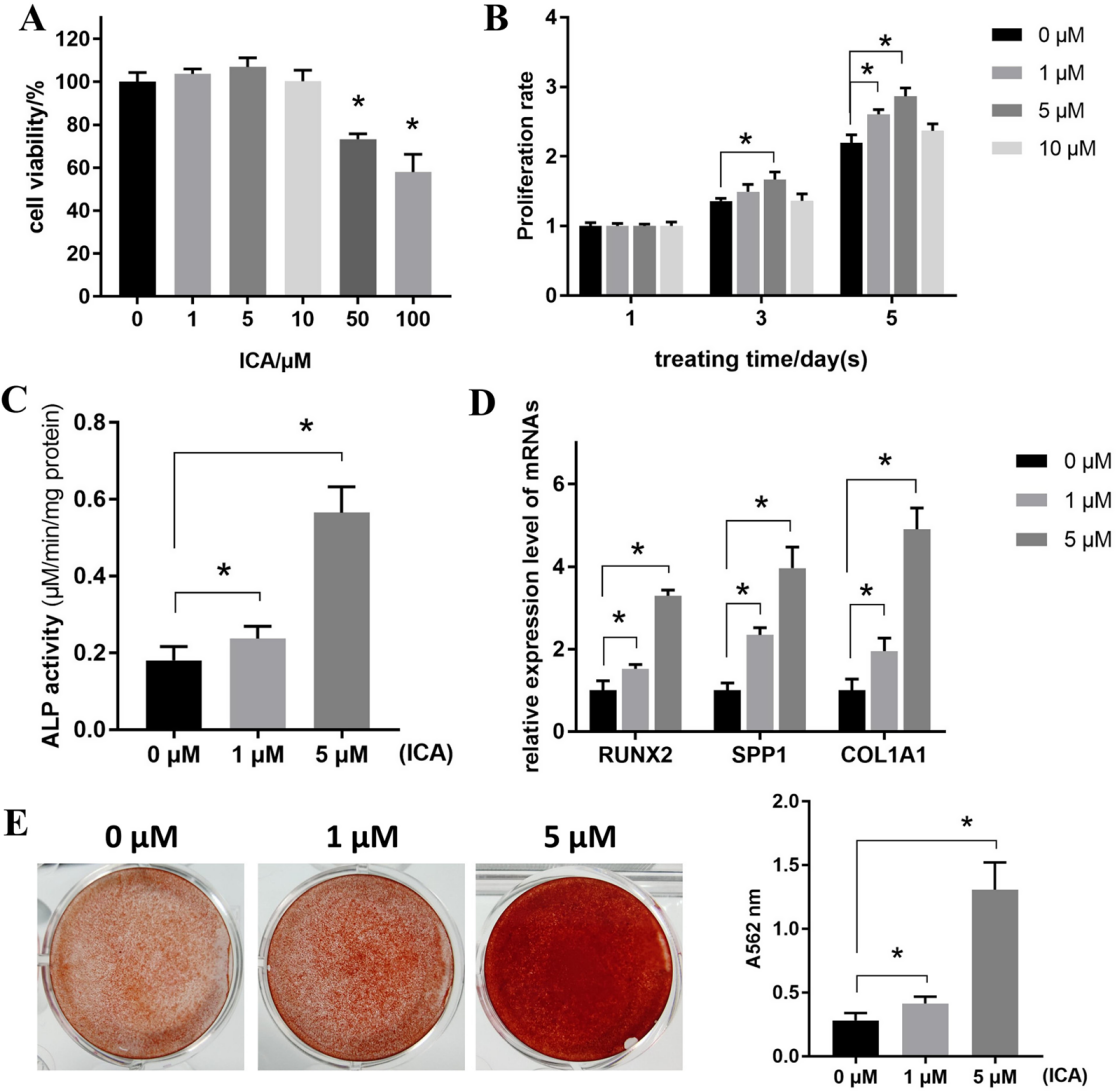
ICA promotes the proliferation and osteogenic differentiation of BMSCs. **A** BMSCs were treated with ICA (0, 1, 5, 10, 50, and 100 μM) for 48 h, and the MTT assay was performed to determine cell viability. **B** BMSCs were with treated ICA (0, 1, 5, and 10 μM), and the MTT assay was performed to determine proliferation rate at different treating time points (1, 3, and 5 d). **C** BMSCs were induced for 7 d with the addition of ICA (0, 1, and 5 μM), and ALP activity was detected. **D** The expression levels of osteogenic markers RUNX2, SPP1, and COL1A1 were measured using qPCR after the BMSCs were induced for 7 d with ICA (0, 1, and 5 μM). **E** Following induction for 21 d in the presence of ICA (0, 1, and 5 μM), mineralization levels were detected using ARS staining

To confirm the appropriate treatment dose of ICA, its cytotoxicity was determined first. As shown in Fig. 2A, lower doses (1–10 μM) of ICA were nontoxic, whereas higher doses (50 and 100 μM) of ICA significantly inhibited the viability of BMSCs. Therefore, lower doses were selected for the following assays. The proliferation results indicated that 1 and 5 μM ICA significantly promoted the growth of BMSCs, and the 5 μM group showed a higher proliferative promotion than the 1 μM group (Fig. 2B). The 10 μM dose seemingly promoted the proliferation of BMSCs, but no significance was detected; therefore, the dose was eliminated in the following assays. After induction with ICA, the osteogenic differentiation of BMSCs was significantly enhanced with higher levels of ALP, RUNX2, SPP1, and COL1A1, and increased mineralization levels were detected (Fig. 2C–E). In addition, the overall differentiation level in the 5 μM group was notably higher than that in the 1 μM group.

### Identification and bioinformatic analysis of the DEGs in BMSCs treated with ICA

**Fig. 3:**
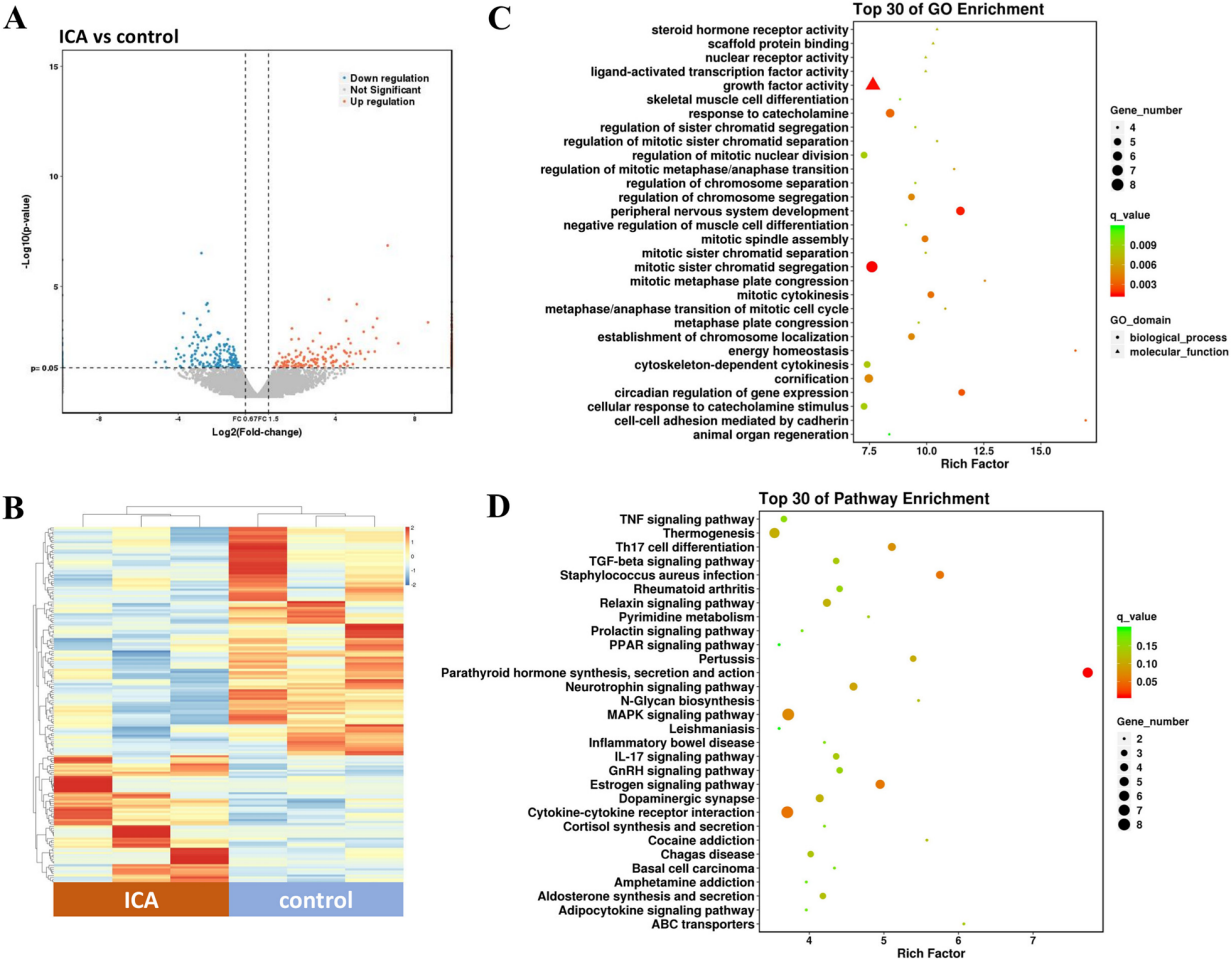
Identification and bioinformatic analysis of the DEGs in BMSCs treated with ICA. **A** The volcano map of DEGs screened in BMSCs treated with 5 μM ICA for 3 d under the thresholds of |FC| value > 2 and p value < 0.05. Red and blue dots indicate upregulated and downregulated genes, respectively. **B** The heap-map of DEGs in each sample. A line indicates a gene and a column indicates a sample. Red and blue indicate upregulation and downregulation, respectively. **C** Top 30 analyses of GO enrichment. The size of circle/triangle indicates the number of genes, and the circle/triangle indicates biological process/molecular function. **D** Top 30 analyses of KEGG pathway enrichment. The size of the circle indicates the number of genes

To investigate the potential mechanism of ICA, the gene expression of BMSCs treated with 5 μM ICA was analyzed using RNA sequencing. Under the thresholds of |FC| value > 2 and *p* < 0.05, 62 upregulated and 111 downregulated DEGs were screened in the ICA group compared to the control group (Fig. 3A, Additional file 1: Table S1). The gene expression profiles of the samples in each group were less consistent, as the BMSCs were isolated from three independent patients instead of three repeats (Fig. 3B).

GO enrichment analysis indicated that several of the screened DEGs were enriched in mitosis-related processes, including metaphase/anaphase transition of the mitotic cell cycle, mitotic cytokinesis, regulation of sister chromatid segregation, and regulation of mitotic nuclear division (Fig. 3C). Therefore, DEGs enriched in these processes were closely associated with the upregulated proliferation of BMSCs stimulated by ICA. KEGG pathway analysis showed that the DEGs were involved in various pathways, including the TGF-beta, estrogen, MAPK, and PPAR signaling pathways, as well as in some diseases (for example, rheumatoid arthritis and inflammatory bowel disease; Fig. 3D).

### Screening and verification of the candidate DEGs

**Fig. 4:**
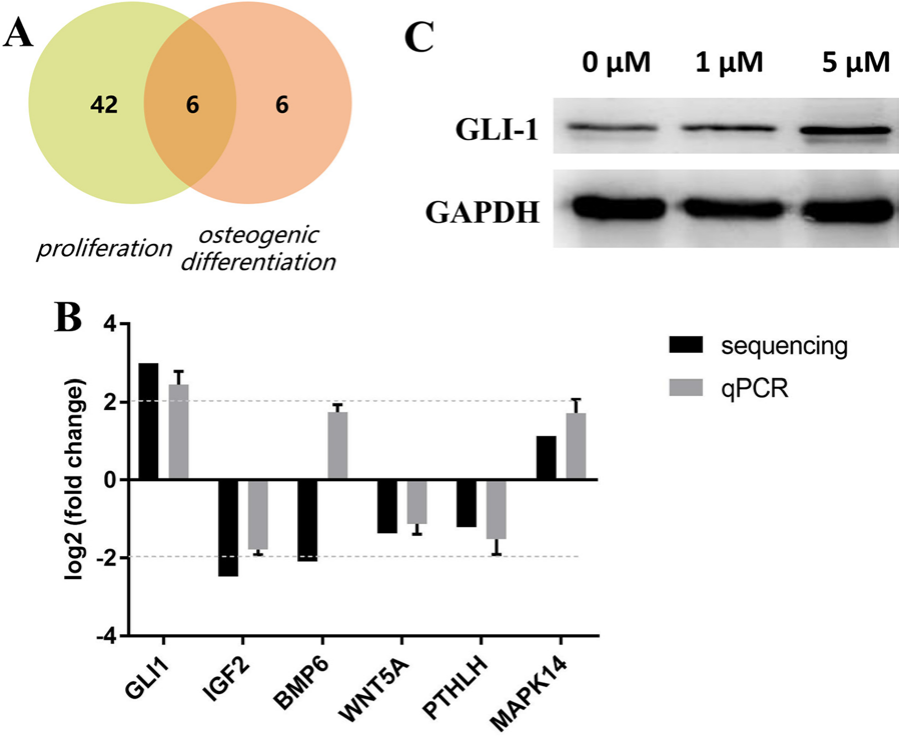
Screening and verification of the candidate DEGs. **A** Venn diagram analysis of overlapping DEGs enriched in proliferation- and osteogenic differentiation-related processes. **B** qPCR detection of the expression fold change of six candidate DEGs in BMSCs treated with 5 μM ICA (vs. cells without ICA treatment). **C** Western blotting was performed to quantify the protein level of GLI-1 in BMSCs treated with ICA (0, 1, and 5 μM) for 48 h

The top 30 GO and KEGG analyses provided some proliferation-related evidence, but osteogenic differentiation-related insights were limited. To screen DEGs involved in both phenotypes, proliferation and osteogenic differentiation, keywords division, proliferation, mitotic aspects, osteoblast activity, bone mineralization, and ossification were used to screen relevant processes. Nine major processes were obtained, and DEGs in some processes overlapped (Table 2). Further analysis indicated that 48 DEGs were enriched in the proliferation phenotype, and 12 DEGs were involved in osteogenic differentiation. Venn diagram analysis showed that six overlapping DEGs (GLI-1, IGF2, BMP6, WNT5A, PTHLH, and MAPK14) were screened in the two phenotypes (Fig. 4A), and their expression levels are listed in Table 3. qPCR validation determined that the FC alterations of five DEGs were consistent with the sequencing results, and GLI-1 exhibited the highest |log2FC| value (Fig. 4B). Western blotting was also performed to further verify the expression of GLI-1, and its protein was enhanced after ICA treatment, positively correlating with the dose of ICA (Fig. 4C).

**Table 2:** Genes enriched in proliferation- and osteogenic differentiation-related phenotypes

Phenotype	Phenotype-related keywords	GO_ID	GO_term	Type	diff_gene_number	diff_gene_list	rich_factor	P value
Proliferation	Division	GO:0140014	Mitotic nuclear division	Biological_process	11	CDC20, MYBL2, MKI67, PLK1, UBE2C, CHMP1A, CDCA5, DLGAP5, CHMP1B, MIS12, IGF2	5.60	2.71E-06
		GO:0051301	Cell division	Biological_process	15	AL358472.7, KIF20A, SFN, SAPCD2, CDCA5, TXNIP, CDC20, PLK1, UBE2C, CHMP1A, CHMP1B, CABLES1, MIS12, PDGFD, IGF2	3.59	1.86E-05
	Proliferation	GO:0008283	Cell population proliferation	Biological_process	37	EPCAM, NGFR, GLI1, SAPCD2, EFNB1, TXNIP, MAPK14, MKI67, CLDN1, TIMP2, CDC20, SFN, EMC10, PPARGC1A, RERG, CSF3, IGF2, AKIRIN1, WNT5A, RORA, PTPRU, BMP6, BDNF, PDPN, BCL2L2, EDNRB, NR4A1, PTHLH, GPR183, NUDT16, IL23A, PRDM1, EGR1, PDGFD, TGM2, NR4A3, IL11	2.57	4.11E-07
	Mitotic	GO:0000278	Mitotic cell cycle	Biological_process	16	UBE2C, MYBL2, MKI67, CDCA5, CDC20, TIMP2, PLK1, CHMP1A, AL358472.7, KIF20A, SFN, SAPCD2, DLGAP5, IGF2, CHMP1B, MIS12	2.15	3.71E-03
Osteogenic differentiation	Osteoblast	GO:0045667	Regulation of osteoblast differentiation	Biological_process	5	GLI1, ATF4, FBN2, BMP6, RANBP3L	5.41	8.79E-04
		GO:0001649	Osteoblast differentiation	Biological_process	7	ATF4, GLI1, RANBP3L, IGF2, BMP6, FBN2, PTHLH	4.32	6.19E-04
	Bone/mineralization	GO:0030282	Bone mineralization	Biological_process	5	ATF4, ENPP1, PTHLH, FBN2, BMP6	6.38	3.78E-04
	Ossification	GO:0030278	Regulation of ossification	Biological_process	5	MAPK14, WNT5A, BMP6, FBN2, ENPP1	6.33	3.94E-04
		GO:0001503	Ossification	Biological_process	12	ATF4, GLI1, MAPK14, ENPP1, FBN2, BMP8B, PTHLH, BMP6, WNT5A, IGF2, BMP8A, RANBP3L	4.23	2.06E-05

**Table 3:** The expression alteration of six overlapping DEGs

	Gene id	Gene name	Gene description	log2FC	log2FC abs	FC abs	*p *Value	Up/down	Locus
1	ENSG00000111087	GLI1	GLI family zinc finger 1	2.98	2.98	7.90	0.034	UP	12:57,459,785–57,472,268
2	ENSG00000167244	IGF2	insulin like growth factor 2	-2.47	2.47	5.53	0.002	DOWN	11:2,129,112–2,141,238
3	ENSG00000153162	BMP6	bone morphogenetic protein 6	-2.09	2.09	4.26	0.012	DOWN	6:7,726,099–7,881,728
4	ENSG00000114251	WNT5A	Wnt family member 5A	-1.36	1.36	2.56	0.020	DOWN	3:55,465,715–55,490,539
5	ENSG00000087494	PTHLH	parathyroid hormone like hormone	-1.21	1.21	2.31	0.042	DOWN	12:27,958,084–27,972,733
6	ENSG00000112062	MAPK14	mitogen-activated protein kinase 14	1.14	1.14	2.20	0.011	UP	6:36,027,677–36,111,236

DEGs: differentially expressed genes; FC: fold change.

### Overexpression of GLI-1 promotes the proliferation and osteogenic differentiation of BMSCs

**Fig. 5:**
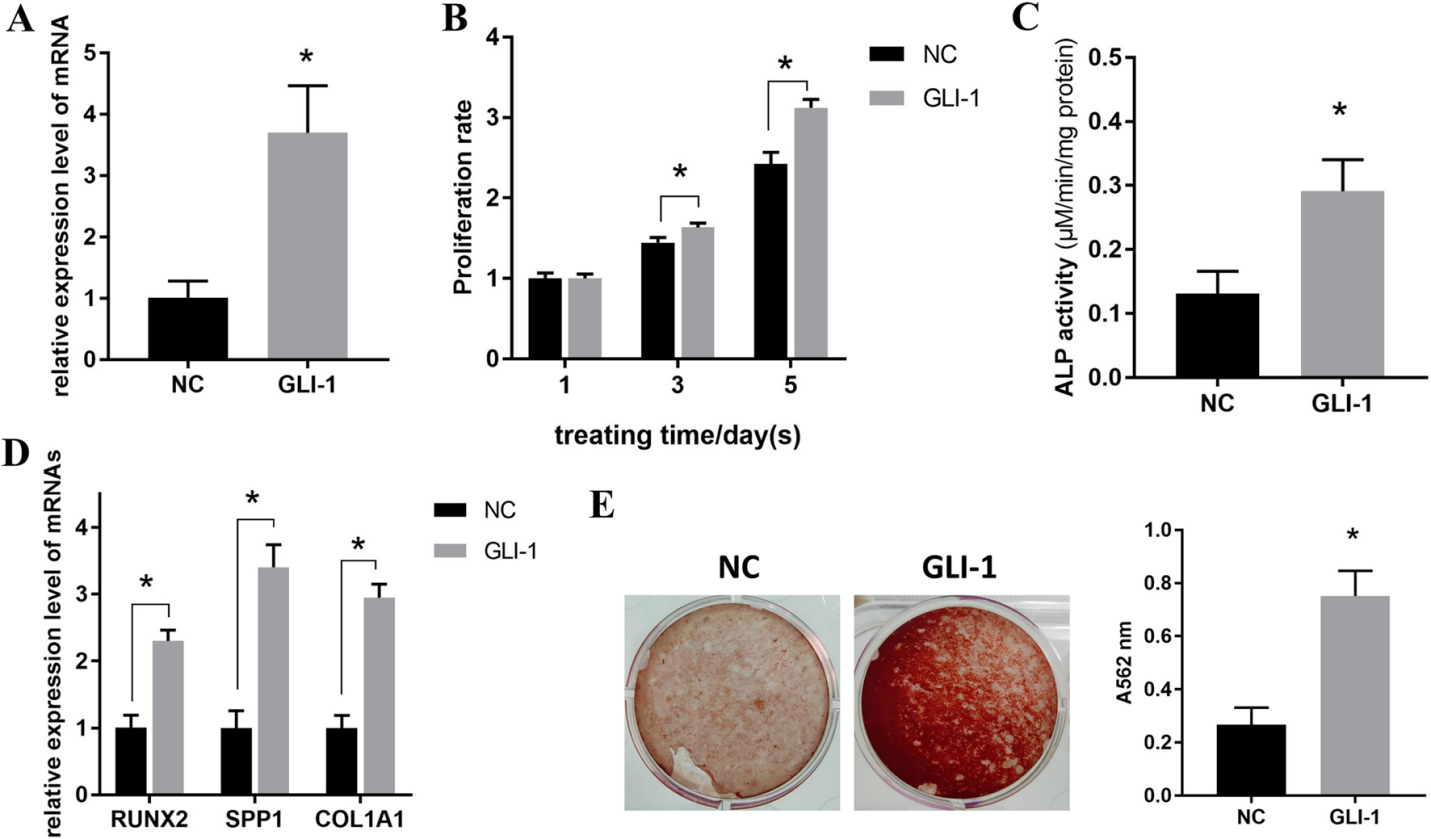
Overexpression of GLI-1 promotes the proliferation and osteogenic differentiation of BMSCs. **A** BMSCs were transfected with GLI-1 overexpression plasmid, and qPCR was performed to determine the mRNA expression level of GLI-1. **B** The MTT assay was performed to determine the proliferation rate at different treating time points (1, 3, and 5 d) after upregulation of GLI-1 in BMSCs. (**C–E**) BMSCs overexpressed with GLI-1 were induced for 7 d, and ALP activity **C** and mRNA level of osteogenic markers **D** were detected using a kit and qPCR, respectively; after induction for an additional 14 d, mineralization level was determined using ARS staining (**E**)

Next, we focused on the role of GLI-1 in the phenotypes of BMSCs. After cells were transfected with the overexpression plasmid, qPCR detection revealed that its expression level was significantly increased compared to that of the NC group (Fig. 5A). The MTT assay results indicated that the increase in GLI-1 promoted the proliferation ability of BMSCs (Fig. 5B). In addition, the osteogenic makers ALP, RUNX2, SPP1, and COL1A1, as well as the mineralization level, were increased following upregulation of GLI-1 (Fig. 5C–E), indicating that GLI-1 enhanced the osteogenic potency of BMSCs.

### Knockdown of GLI-1 suppresses the positive effects of ICA on the proliferation and osteogenic differentiation of BMSCs

**Fig. 6:**
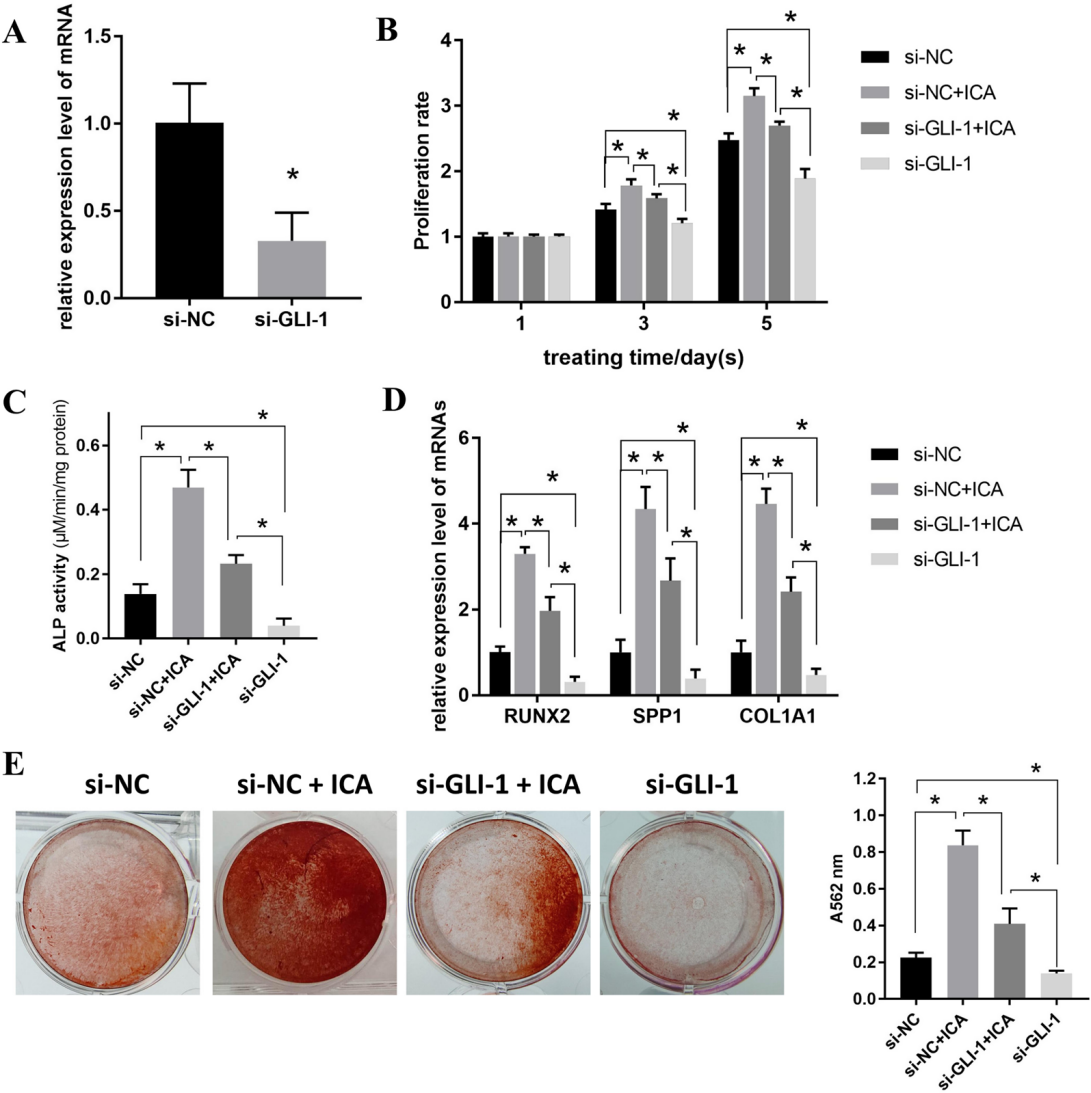
Knockdown of GLI-1 suppresses the positive effects of ICA on the proliferation and osteogenic differentiation of BMSCs. **A** qPCR was used to determine the mRNA expression level of GLI-1 after transfection of siRNA targeting GLI-1 in BMSCs. **B** The MTT assay was performed to determine the proliferation rate at different treating time points (1, 3, and 5 d) after downregulating GLI-1 in BMSCs treated with or without 5 μM ICA. (**C–E**) BMSCs silenced with GLI-1 were induced for 7 d with or without 5 μM ICA, and ALP activity **C** and mRNA level of osteogenic markers **D** were determined using a kit and qPCR, respectively; after induction for an additional 14 d, mineralization level was determined using ARS staining (**E**)

As shown in Fig. 6A, the expression level of GLI-1 was reduced by approximately 70% after transfection with siRNA. The proliferation assay results indicated that the knockdown of GLI-1 significantly inhibited the proliferation rate enhanced by ICA (Fig. 6B). Similarly, the osteogenic potential of BMSCs in si-NC + ICA was also weakened after downregulation of GLI-1, with significant decreases in ALP, RUNX2, SPP1, and COL1A1, as well as the mineralization level observed in the si-GLI-1 + ICA group (Fig. 6C–E). The role of GLI-1 downregulation in BMSCs without ICA treatment was also investigated. As expected, the results indicated that knockdown of GLI-1 significantly suppressed the proliferation and osteogenic potency of BMSCs compared to that in the si-NC group (Fig. 6B–E).

## Discussion

Transplantation of MSC has promising applications in regenerative medicine and the treatment of various pathological disorders, including diabetes [21, 22]. Application of autologous cells is more common than allogenic cells, as it may help avoid the risk of immune rejection. However, the self-proliferation and multi-lineage differentiation capabilities are less than optimal when the donors are elderly or have a history of diabetes for decades [23, 24]. Amelioration of the impaired functions of MSCs is of great significance for future autologous transplantation. The present study, for the first time, showed that ICA promoted the proliferation and osteogenic potency of BMSCs derived from OP patients with T2DM and identified the key effector gene GLI-1 of ICA based on sequencing and bioinformatic analyses.

The ICA dose used in the cytotoxicity assay varied from 1 to 100 μM, which was based on several relevant studies. For instance, 1 and 10 μM ICA are both positive for the osteogenic potency of amnion MSC, and 1, 10, and 100 μM ICA are all able to promote the proliferation of amnion MSC [25]. Another study indicated that 20 μM ICA has a better osteogenic stimulating effect on bone marrow BMSC than doses of 10 or 40 μM [26]. The present study found that 5 μM ICA had the best proliferative promoting effect, which became much weaker when the dose was doubled. In addition, with a further increase in ICA dose, the viability of BMSCs was significantly suppressed, which was also reported in other studies [18, 26]. Therefore, the dose with the best stimulating proliferation effect was selected.

RUNX2 is a critical transcription factor that plays an important role in the initiation of osteogenic differentiation and the expression of genes (for example, ALP, and SPP1) related to bone formation [27, 28]. In the later stage, the extracellular matrix is gradually mineralized owing to calcium deposition, ultimately forming bone nodules [29]. The present study found that ICA treatment induced significant increases in RUNX2, ALP, and SPP1 after 7-d induction, and an enhancement of mineralization level in the later stage (21-d induction), thus confirming a positive effect of ICA on the osteogenic differentiation of BMSCs. The ideal dose (5 μM) was different from those (1 or 20 μM) in previous studies [25, 26], and this may be related to the properties of MSCs.

RNA sequencing identified several DEGs after ICA treatment, and bioinformatic analysis indicated that 48 DEGs were enriched in proliferation-related processes, much higher than 12 DEGs in osteogenic differentiation-related processes. This difference in number might be because BMSCs were directly simulated with ICA, not induced in supplementation with ICA. The present study aimed to screen for DEGs involved in proliferation and osteogenic differentiation; hence, six overlapped genes were obtained. GLI-1, with the highest validated |log2FC| value, was selected for further investigation.

GLI-1, also known as glioma-associated homologue-1, was first discovered in 1987 as an amplified gene in glioblastoma multiforme [20]. It encodes a zinc finger transcription factor, which is a member of the Kruppel family of zinc finger proteins. GLI-1 is a key nuclear mediator in hedgehog signaling, thus playing important roles in embryonic development and tumorigenesis. Studies indicate that GLI-1 + cells originating from periarterial cells and suture mesenchyme are both MSCs/progenitor cells, responsible for tissue/organ development and injury repair in the craniofacial region [30, 31]. In addition, Gli1 + cells contribute to nearly all osteoblasts during postnatal condylar development and bone fracture healing [32, 33]. These reports indicate that GLI-1 + cells have the properties of MSCs, including self-renewal and multi-lineage differentiation capabilities, as well as the promising repair ability. Therefore, the higher number of GLI-1 + cells suggested higher proliferation and osteogenic potency. Similarly, the present study found that overexpression of GLI-1 enhanced the proliferation and osteogenic differentiation of BMSCs, whereas downregulation of its level caused the opposite results. Moreover, we also found that knockdown of GLI-1 weakened the positive effect of ICA in BMSC, thereby confirming that ICA exerted its role by upregulating GLI-1 expression.qPCR validation indicated that the expression level of BMP6 also increased after BMSCs were treated with ICA. It is well known that BMP6 exerts a positive role in the process of osteogenic differentiation [34]. Interestingly, ICA can induce the upregulation of BMP6 in MG-63 cells [35]. Therefore, BMP6 may be another effector gene of ICA that requires further investigation. Another notable gene was WNT5A, which decreased after ICA stimulation. It is reported that WNT5A plays a promoting role in the osteogenic differentiation of MSCs [36, 37]. The downregulation of WNT5A in BMSCs treated with ICA indicated the inhibition of osteogenic potency to some extent. This was not contradictory to the positive effect of ICA, as the inhibitory role of WNT5A could be offset by the promoting roles of other genes.

KEGG pathway enrichment analysis showed that DEGs were enriched in various pathways, including the MAPK signaling pathway. Studies indicate that the MAPK pathway plays an important role in the proliferation and osteogenic differentiation of MSCs [38, 39]. It has been reported that the upregulation of Sonic Hedgehog (SHH)-induced GLI-1 and MAPK-ERK is involved in the differentiation process of pre-osteoblasts [40]. Additionally, selective inhibition of p38 MAPK significantly attenuates the upregulation of GLI-1 induced by SHH [41]. Therefore, there might be a relationship between GLI-1 and the MAPK pathway, which requires further investigation. Notably, we found that activating transcription factor 4 (ATF4), which is enriched in the MAPK pathway, was also involved in various osteogenic differentiation-relevant GO terms. It has been reported that ATF4 plays positive regulatory roles in the osteogenic differentiation of periodontal ligament stem cells [42] and valvular interstitial cells [43], as well as in bone information [44]. The MAPK pathway is also one of the major pathways for ATF4 regulation of osteogenic differentiation [45, 46]. Therefore, the upregulated ATF4 in the ICA group may be another potential target for ICA exerting its pro-osteogenic effect, as knockdown of GLI-1 could not completely inhibit ICA’s effect of ICA.

The present study found that ICA can promote the proliferation and osteogenic potency of BMSCs and proved that the upregulation of GLI-1 was responsible for the positive effect of ICA. The study provides a potential new molecule to ameliorate the impaired function of BMSCs derived from OP patients with T2DM, which is of some positive meaning for effective autologous transplantation in future.

## Supplementary Information

**Additional file 1. Table S1 **The expression of 173DEGs in BMSCs treated with ICA.

## Data Availability

The data analyzed during the study are available from the corresponding author on reasonable request.
